# COVID-19 Associated Rhino-Orbital Mucormycosis Complicated by Gangrenous and Bone Necrosis—A Case Report from Honduras

**DOI:** 10.3390/vaccines9080826

**Published:** 2021-07-27

**Authors:** Elsa Yolanda Palou, María Auxiliadora Ramos, Emec Cherenfant, Adoni Duarte, Itzel Carolina Fuentes-Barahona, Lysien I. Zambrano, Fausto Muñoz-Lara, Sandra Aracely Montoya-Ramirez, Alex Francisco Cardona-Ortiz, Jorge Alberto Valle-Reconco, Juan J. Montenegro-Idrogo, D. Katterine Bonilla-Aldana, Alberto E. Paniz-Mondolfi, Alfonso J. Rodriguez-Morales

**Affiliations:** 1 Department of Internal Medicine, Faculty of Medical Sciences, Universidad Nacional Autónoma de Honduras, Tegucigalpa 11101, Honduras; elsa.palou@unah.edu.hn (E.Y.P.); faustomunoz@doctor.com (F.M.-L.); 2 Department of Internal Medicine, Hospital Escuela, Tegucigalpa 11101, Honduras; mariaramosynestroza@yahoo.com; 3 Universidad Nacional Autónoma de Honduras, Tegucigalpa 11101, Honduras; investigacion_fcm@unah.edu.hn; 4 Department of Morphological Sciences, Faculty of Medical Sciences, Universidad Nacional Autónoma de Honduras, Tegucigalpa 11101, Honduras; adoni.duarte@unah.edu.hn; 5 Faculty of Medical Sciences, Universidad Nacional Autónoma de Honduras, Tegucigalpa 11101, Honduras; ixcarol@hotmail.com; 6 Department of Gynecology and Obstetrics, Hospital Escuela, Tegucigalpa 11101, Honduras; 7 Unit of Scientific Research (UIC), Faculty of Medical Sciences, Universidad Nacional Autónoma de Honduras, Tegucigalpa 11101, Honduras; lysien.zambrano@unah.edu.hn; 8 Latin American Network of Coronavirus Disease 2019 Research (LANCOVID), Pereira, Risaralda 660003, Colombia; jmontenegroi@cientifica.edu.pe (J.J.M.-I.); diana.bonilla@uam.edu.co (D.K.B.-A.); alberto.Paniz-mondolfi@mountsinai.org (A.E.P.-M.); 9 Department of Mycology, Hospital Escuela, Tegucigalpa 11101, Honduras; montoyasandra62@yahoo.es; 10 Post-Graduate Internal Medicine, Faculty of Medical Sciences, Universidad Nacional Autónoma de Honduras, Tegucigalpa 11101, Honduras; alex_3363007@yahoo.com; 11 Deanship, Facultad de Ciencias Médicas, Universidad Nacional Autónoma de Honduras, Tegucigalpa 11101, Honduras; jorge_valle@unah.edu.hn; 12 Faculty of Health Sciences, Universidad Cientifica del Sur, Lima 15046, Peru; 13 Infectious and Tropical Diseases Service, Hospital Nacional Dos de Mayo, Lima 15072, Peru; 14 Semillero de Investigación en Zoonosis (SIZOO), Grupo de Investigación GISCA, Fundación Universitaria Autónoma de las Américas, Sede Pereira, Pereira 660003, Colombia; 15 Laboratory of Microbiology, Department of Pathology, Molecular and Cell-Based Medicine, Icahn School of Medicine at Mount Sinai, New York, NY 10029-6574, USA; 16 Instituto de Investigaciones Biomédicas IDB/Incubadora Venezolana de la Ciencia, Barquisimeto 3001, Venezuela; 17 Grupo de Investigación Biomedicina, Faculty of Medicine, Fundación Universitaria Autónoma de las Americas, Pereira, Risaralda 660003, Colombia; 18 Master of Clinical Epidemiology and Biostatistics, Universidad Cientifica del Sur, Lima 15046, Peru

**Keywords:** SARS-CoV-2, COVID-19, mucormycosis, mucorales, coinfection, opportunistic, honduras

## Abstract

Background: Mucormycosis is a life-threatening invasive fungal infection most commonly observed in immunocompromised patients. Throughout the COVID-19 pandemic, a growing number of *Mucorales* associated infections, now termed COVID-19 associated mucormycosis (CAM), have been reported. Despite an increase in fatality reports, no cases of rhino-orbital CAM complicated with gangrenous bone necrosis have been described in the literature to date. Case: A 56-year-old male with a recent COVID-19 diagnosis developed rhino-orbital mucormycosis after 22 days of treatment with dexamethasone. Cultures and histopathological assessment of tissue biopsy confirmed the diagnosis. The patient survived after treatment with amphotericin B. Conclusions: Mucormycosis is an invasive fungal infection affecting mostly immunocompromised patients. Along with the COVID-19 pandemic, the inappropriate use of steroids, in addition to concurrent risk factors, such as diabetes, has led to an increase in the occurrence of these devastating mycoses, leading to the development of severe presentations and complications, as observed in many cases. Early diagnosis and prompt treatment are crucial in order to avoid dissemination and fatal outcomes.

## 1. Introduction

Mucormycosis is a fairly rare but life-threatening invasive fungal infection caused by various genera and species of the order Mucorales, with six families being the most common *Mucoraceae* affecting immunocompromised patients [1]. Mucormycosis most commonly occurs in patients with underlying host defense defects and increased serum iron availability, although sporadically, immunocompetent hosts may be affected [1,2,3]. Mucormycosis is also an important emerging condition among patients with haematological malignancies, allogeneic stem cell transplantation, and diabetes mellitus [4]. Historically, the inappropriate use of steroids has been linked with the development of this invasive fungal infection [5,6], particularly if associated with any of the abovementioned risk factors [7,8]. Moreover, inappropriate steroid use may trigger concurrent invasive fungal infections, such as concomitant mucormycosis and aspergillosis [9].

Early in the coronavirus disease 2019 (COVID-19) pandemic in 2020, a pressing need to develop and repurpose effective therapeutic approaches to manage severe acute respiratory syndrome coronavirus 2 (SARS-CoV-2) infection led to consideration of a myriad of drug options. Multiple clinical trials were developed, including those assessing the role of steroids, particularly for severe clinical cases. One of these trails, the RECOVERY trial, showed a marked reduction in mortality at day 28 after randomization among those receiving either invasive mechanical ventilation or oxygen supplementation alone [10]. Unfortunately, as with other drugs, such as chloroquine, there has been an inappropriate use of these drugs in clinical scenarios where only marginal or no benefits have been achieved [11,12,13,14]. Likely associated to an inappropriate use of steroids, a growing number of mucormycosis among patients with COVID-19, now catalogued as COVID-19 associated mucormycosis (CAM), have been reported in multiple countries across the globe [15], including also some Latin American countries such as Brazil and Mexico [16,17].

While most reports and fatality cases correspond to rhino-orbital and pulmonary infection [16,17,18,19], to the best of our knowledge, no other previous cases of rhino-orbital CAM, complicated by gangrenous bone necrosis, has been reported in the literature to date. 

## 2. Case Report

A 56-year-old male from San Pedro Sula, Cortes, Honduras, with no significant past medical history, was assessed at the hospital in the clinical triage due to respiratory symptoms including dyspnea and fever in his town on 31 December 2020, although an antibody testing was negative, he was suspected to have a moderate COVID-19 and given his symptoms persistence was diagnosed by SARS-CoV-2 antigen testing as RT-PCR was not available at that time during a medical brigade. On 8 January 2021, a thoracic CT-scan showed peripheral ground-glass opacities (35%). He received dexamethasone (8 mg/day) for 22 days as outpatient, having CT-scan controls on 23 and 29 January 2021, showing clinical and radiological improvement. 

A month later, on 31 January 2021, he was admitted to the Mario Catarino Rivas Hospital in San Pedro Sula, after exhibiting inflammatory changes in his right hemiface accompanied by intense headache, diplopia, decreased visual acuity in the right eye, amaurosis, and signs of right periorbital cellulitis. At that time, the diagnosis of diabetes mellitus was suspected due to hyperglycemia, later ruled out (normal glycemia and HbA_1C_) and was diagnosed with hypertension. He was immediately placed on antibiotics, enoxaparin, furosemide, ranitidine, amlodipine, bisoprolol, enalapril, and tramadol. 

**Figure 1:**
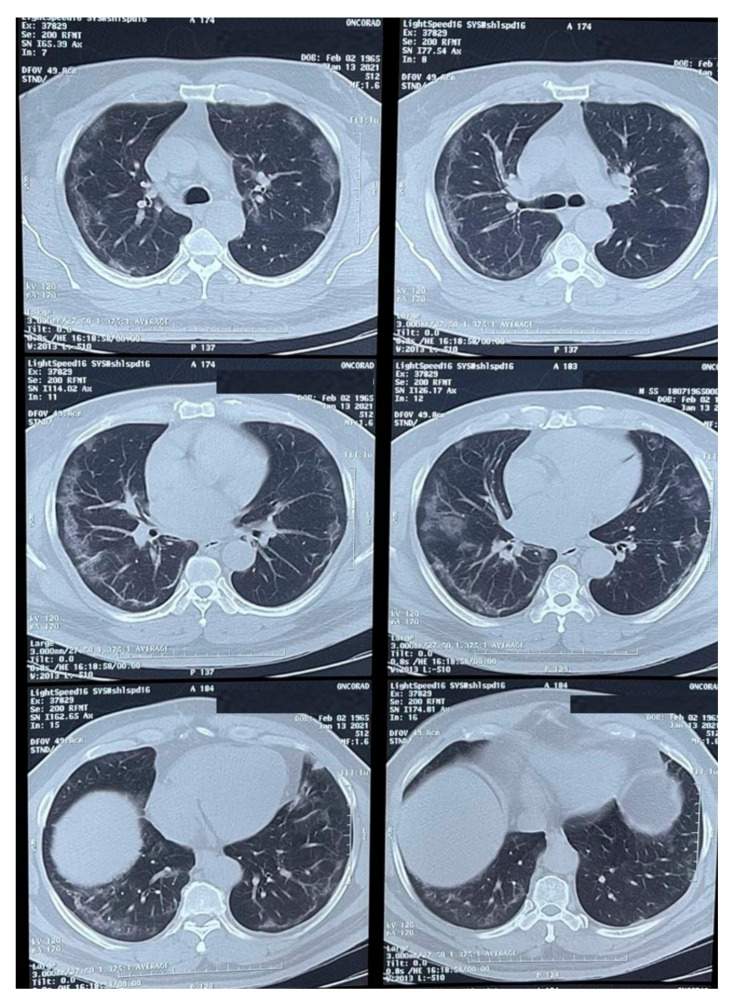
CT-scan of the thorax showing peripheral ground-glass opacities with a partial occupation and consolidations.

Physical examination revealed an ulcerated lesion in the oral mucosa. A scraping from the lesion was collected and sent for potassium hydroxide (KOH) preparation and bacterial / fungal cultures. Multiple imaging studies were performed, including a thoracic CT-scan (Figure 1) that showed peripheral ground-glass opacities with a partial pleural effusion and consolidation. 

**Figure 2:**
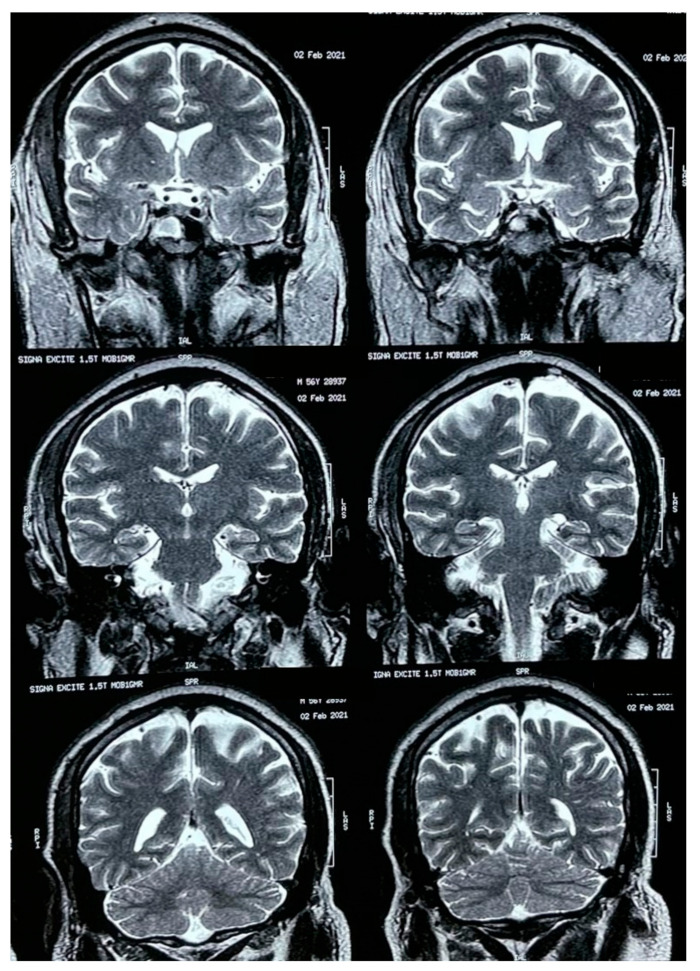
Brain MRI.

**Figure 3:**
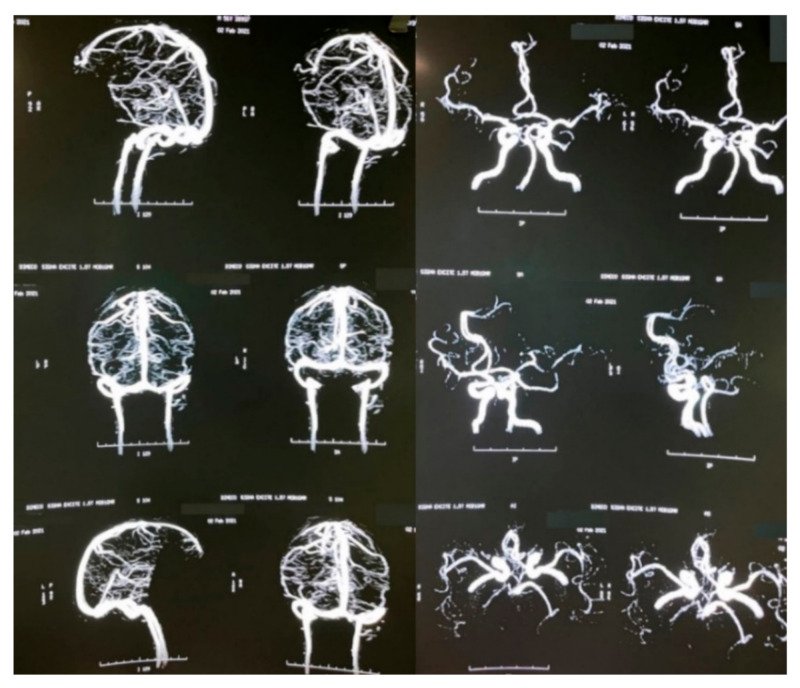
Brain angiography.

**Figure 4:**
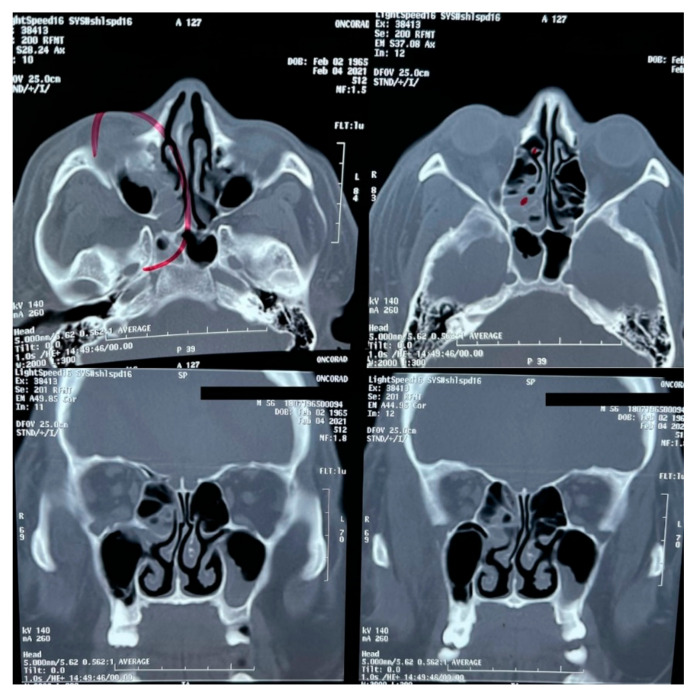
CT-scan of the paranasal sinuses.

A brain MRI was performed a couple of weeks later showing no abnormalities on brain parenchyma nor signs of infarction (Figure 2). Also, a cerebral angiography (Figure 3) and a CT-scan of the paranasal sinuses were performed. The cerebral angiography was unremarkable. Paranasal sinuses showed marked oedema of soft tissues with mucosal thickening on the right side in close vicinity to the right orbit. The sphenoid sinus showed mucosal thickening of the right side with approximately 70% obstruction of the ethmoid cells. The superior turbinate of the right side was involved as well. Destruction of the superior turbinate region was also observed. Occupation of the maxillary sinus on the left side related to sinusitis on the left side was also evident (Figure 4).

**Figure 5:**
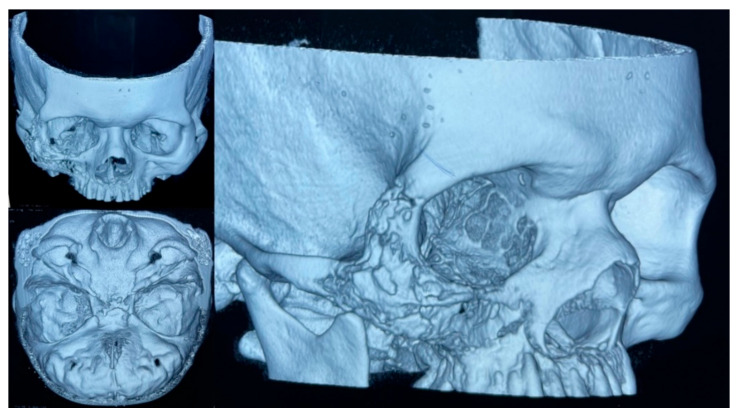
A 3D multislice CT-scan of the head showed the mucormycosis associated with bone destruction.

On 10 February 2021, an initial biopsy of the lesion was performed showing non-specific tissue repair and inflammatory changes. Despite antibiotic therapy, the patient did not improve and inflammatory changes in the right hemiface persisted, although e was discharged on 17 February 2021. Upon close examination, as an outpatient, purulent secretion through the nostrils and oral cavity was noted, and a second biopsy of the lesion in the palate was taken. Histopathological changes consistent with acute and chronic osteomyelitis were reported and a diagnosis of mucormycosis was suspected. A 3D multislice head CT-scan of the head showed significant destruction of the right upper maxillary bone, associated with lytic lesions of the floor of the right orbit and ipsilateral wall of the maxillary sinus (Figure 5). There was presence of inflammatory changes involving the maxillary sinuses, ethmoid cells, and frontal sinus of the right side.

**Figure 6:**
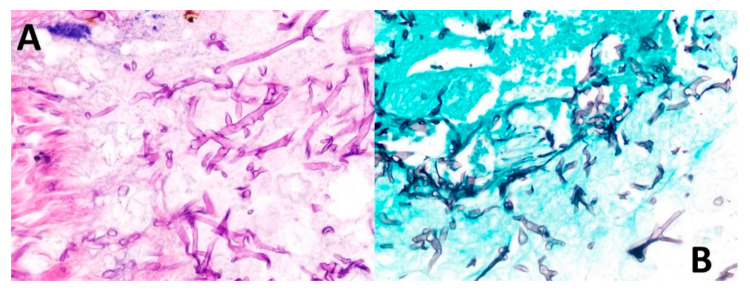
Biopsy findings. (**A**) Routine staining with hematoxylin and eosin shows broad ribbon-shaped hyphae without marked septa, 5–15 microns in diameter and irregular angled branching ranging from 45 to 90 degrees. (**B**) Grocott’s methenamine stain permeates the walls of fungal cells due to its high density of polysaccharides, retaining more aldehyde groups by oxidation of chromic acid and highlighting the hyphal structures.

On 13 May 2021, the patient was scheduled for reconstructive surgery in a private hospital in Tegucigalpa. During surgery, part of the right maxilla, malar bone and orbit floor were removed, along with tissue debridement. Finally, removed tissues were sent for histopathological studies. Final pathological revealed mucormycosis with gangrenous necrosis and secondary osteonecrosis (Figure 6) and was hospitalized on 16 May 2021 in Tegucigalpa, the capital of Honduras. Routine staining with hematoxylin and eosin (H&E) showed broad ribbon-shaped hyphae without marked septa, 5–15 microns in diameter and irregular angled branching ranging from 45 to 90 degrees (Figure 6). Morphological diagnosis was confirmed by Grocott’s methenamine silver, which highlighted characteristic hyphal structures (Figure 6).

**Figure 7:**
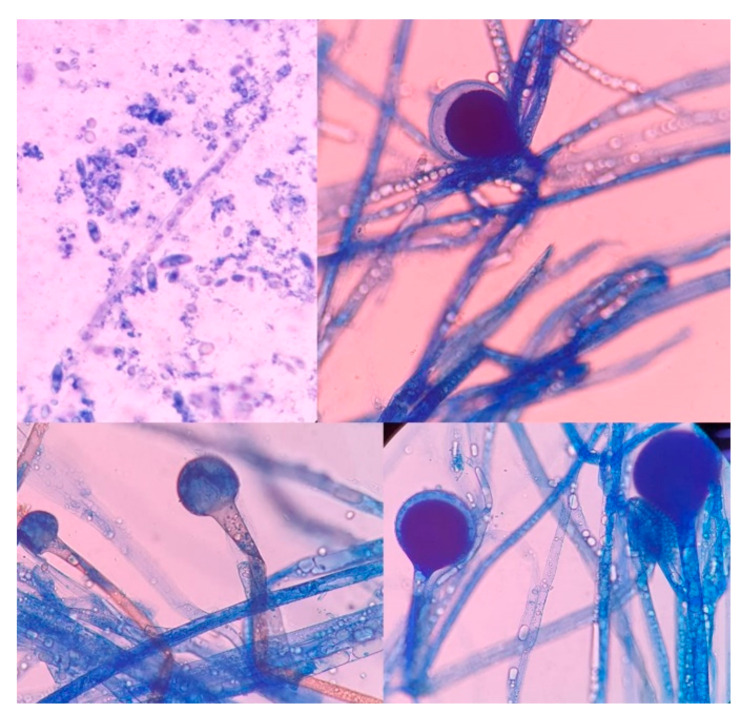
High-power photomicrograph showing the sporangia (Lactophenol cotton blue stain). The wall of the sporangium dissolves on maturity, exposing the spores.

**Figure 8:**
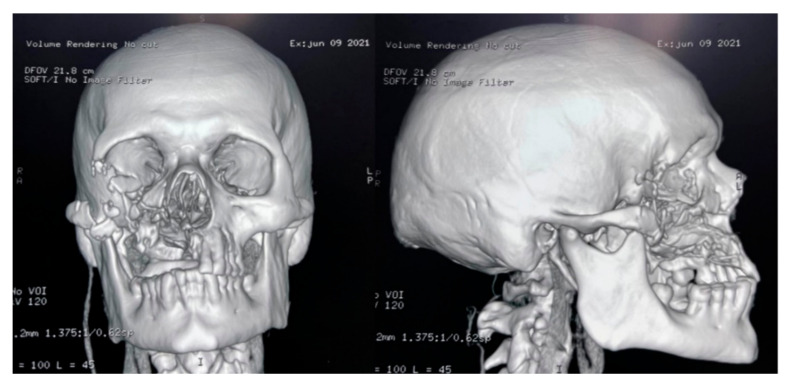
CT-scan of the head after the surgery.

The culture, performed at the Hospital Escuela, in Tegucigalpa, yielded transparent grey colonies with broad and practically non-septate hyphae at microscopy (Figure 7). Long branched sporangiophores deployed of rhizoid structures allowed to identify morphologically as *Mucor* (Figure 7). The patient was initiated on antifungal treatment with amphotericin B (deoxycholate), with 1000 mg (1 g) of cumulative dose. After surgery, on 9 June 2021, an open lesion in the vestibular mucogingival fold of the upper jaw, without signs of bacterial infection, remained patent (Figure 8). The patient was then switched to liposomal amphotericin B with significant clinical improvement on 12 June 2021 when reached a cumulative dose of 2000 mg (2 g). On 16 June 2021, the patient presented a fever (39–40 °C) whilst he was with a central venous catheter (CVC), receiving imipenem and vancomycin. The CVC was retired, and the device and blood were cultured; a sample from the palate ulcer was also cultured. *Pseudomonas* spp. was yielded from the CVC. *Pseudomonas aeruginosa* was yielded from the palate ulcer. Both isolates were susceptible to ciprofloxacin, imipenem and meropenem. He completed eight days of imipenem before being switched to oral ciprofloxacin. On 22 June 2021, he started oral isavuconazole. At 48 h, good tolerance was observed to this drug, with normal renal and hepatic laboratory tests and normal hemogram and biochemistry. We ruled out diabetes in this hospitalizacion due to normal glucose and HbA_1c_. An assessment by the department of plastic surgery revealed a good evolution of the patient’s recovery. He was discharged on 24 June 2021, in good conditions, continuing at home on oral isavuconazole for four additional weeks to complete two months of treatment on 22 July 2021. A control of samples for bacteria and fungi culture was performed, KOH test was negative.

## 3. Discussion

Mucormycosis represents a complex and challenging clinical entity, both from a diagnostic and management standpoint [1,4]. In many cases, this invasive fungal infection represents a life-threatening condition, especially when the central nervous system (CNS) is compromised [16,17,18,19]. For these reasons, when suspecting mucormycosis, it is important to assess by appropriate CNS imaging (Figure 2 and Figure 3). Nowadays, in context of the COVID-19 pandemic, prevalent risk factors, such as diabetes, especially decompensated [4,5,6,8,17,20], and most importantly the inappropriate use of steroids, has led to an increase of cases similar to the one presented herein [15]. For those patients with known risk factors such as diabetes, malignancies (particularly haematological), steroid use, neutropenia, HIV/AIDS, and immunosuppression in general, mucormycosis should always be considered in the differential diagnosis, in order to deliver prompt treatment [1,15]. Now COVID-19 has joined the list of potential predisposing conditions. Initial systematic reviews have shown that at least 43% of COVID-19 affected patients (95% CI 18–67%) developed lymphopenia [20], which may increase the risk of opportunistic infections, including mucormycosis [21]. In this case from Honduras, the patient was unaware of his risk factors, and shortly after presenting COVID-19 and after a 22-day-course of dexamethasone, he developed this complicated form of mucormycosis, to which he fortunately survived. Sometimes some comorbidities are identified together with the COVID-19 diagnosis.

Mucormycosis associated osteonecrosis has rarely been described in non-COVID-19 patients, with only six cases previously reported in the literature thus far [22,23,24,25,26,27]. In 2005, a 59-year-old diabetic male patient from Taiwan presented with an oral ulcer that extended to the upper maxillary bone accompanied by necrosis involving the hard palate alveolar ridge [25]. In general, maxillary osteonecrosis in patients with rhino-cerebral mucormycosis is uncommon [13]. As in our case, the patient survived. Oftentimes these lesions can be misdiagnosed as malignancies, particularly in elderly patients, which is the reason why biopsy and CT-scans are essential for differentiating such conditions [26]. Occasionally, debridement of extensive bone destruction also involves orbital nucleation. In other instances, the eye can be preserved, although patients can become blind, due to extensive compromise of the orbital floor [24]. Fortunately, for our patient, his eye and vision were preserved. To date, no other cases of mucormycosis with osteonecrosis have been reported in context of COVID-19 infection.

A pathologic hallmark of mucormycosis is the frequent presence of extensive angioinvasion, which results in vessel thrombosis and tissue necrosis [1,4], leading to the development of gangrenous lesions [28,29,30,31,32,33,34,35]. As reported in multiple cases, mucormycosis may affect brain vessels. In general, for central nervous system [36,37] involvement, imaging studies, such as CT-scans, MRI, and angiography, are recommended to rule out neurological compromise and infection, as we did for our case. Although our patient did not exhibit direct neurological affectation, he did present with a constellation of neuro-ophthalmological manifestations, including, headache, diplopia, decreased visual acuity in the right eye, and amaurosis. Recently, a similar case from the United State was reported on a non-COVID-19 patient. This case was a 55-year-old diabetic male who complained of headache, maxillary sinus pain, and diplopia for three days, in whom mucormycosis was later confirmed [36]. Likewise, two cases were reported from Spain in 2019, both patients in their 50s, one diabetic, the other in chronic renal failure, who presented sudden unilateral amaurosis depicting an atypical presentation form of rhino-orbital-cerebral mucormycosis [37].

A multidisciplinary approach is key to managing these patients, as mucormycosis can affect several systems, cutaneous, rhino-cerebral, pulmonary, gastrointestinal, and circulate, leading a significant proportion of patients to fatal outcomes [1]. Recent reports on mucormycosis from India have shown a case-fatality rate of 45.7% (at 12 weeks) for both CAM and non-CAM patients [38]. In Latin America, where COVID-19 arrived almost three months later (March 2020) after the emergence of SARS-CoV-2/COVID-19 in China (December 2019), healthcare practitioners and public health authorities should remain vigilant and prepare for a possible increase in cases of mucormycosis associated with COVID-19 patients. Recently, the Pan-American Health Organization (PAHO) issued an epidemiological alert, calling member states to prepare their health services to respond and mitigate the impact of CAM in the Americas [39]. So far, seven countries in the region, including Honduras, Brazil, Chile, Mexico, Paraguay, Uruguay, and United States, have identified CAM cases [39].

Surgical debridement, antifungal treatment and, if possible, adequate control of underlying risk factors are critical for managing these patients [15,39]. The main antifungal drugs used for treatment of mucormycosis include amphotericin B, isavuconazole, and posaconazole. However, amphotericin B remains the primary drug of choice, with proven efficacy, as seen in our case. Therapeutic responses should be carefully monitored in these patients, even more, considering that it is still not clear if evolution and fatal outcomes would be higher due to other concurrent infections and complications in COVID-19 patients. Updated COVID-19 guidelines [40] should then consider options for diagnosis and management of CAM. Mucormycosis new guidelines should also consider this new scenario [41].

## 4. Conclusions

Although mucormycosis has entered public consciousness in response to an outbreak of cases in India [42], this has classically been concerning invasive fungal infection among immunocompromised patients. With the COVID-19 pandemic, and the inappropriate use of steroids, in addition to concurrent risk factors, such as diabetes, an increase in the occurrence of associated mucormycosis is now being observed, leading to the development of severe presentations and complications, as witnessed in this case. Early diagnosis and prompt treatment are crucial in order to avoid circulation and fatal outcomes.
